# Aclidinium improves exercise endurance, dyspnea, lung hyperinflation, and physical activity in patients with COPD: a randomized, placebo-controlled, crossover trial

**DOI:** 10.1186/1471-2466-14-209

**Published:** 2014-12-23

**Authors:** Kai M Beeh, Henrik Watz, Luis Puente-Maestu, Luis de Teresa, Diana Jarreta, Cynthia Caracta, Esther Garcia Gil, Helgo Magnussen

**Affiliations:** insaf Respiratory Research Institute, Wiesbaden, Germany; Pulmonary Research Institute at LungClinic Grosshansdorf, Airway Research Centre North, German Centre for Lung Research, Woehrendamm 80, D-22927 Grosshansdorf, Germany; Hospital General Universitario Gregorio Marañón-Universidad Complutense, Madrid, Spain; Hospital de San Vicente, Alicante, Spain; AstraZeneca PLC, Barcelona, Spain; Formerly of Forest Research Institute, Forest Laboratories LLC, a subsidiary of Actavis, Jersey City, NJ USA

**Keywords:** Aclidinium, COPD, Exercise endurance, Long-acting muscarinic antagonists, Physical activity

## Abstract

**Background:**

This study evaluated the effects of aclidinium bromide, a long-acting muscarinic antagonist indicated for maintenance treatment of chronic obstructive pulmonary disease (COPD), on exercise endurance, dyspnea, lung hyperinflation, and physical activity.

**Methods:**

In this randomized, double-blind, crossover study, patients with stable COPD and moderate-to-severe airflow limitation received aclidinium 400 μg twice daily or placebo via Genuair^®^/Pressair^®^^a^ for 3 weeks (2-week washout between treatment periods). The primary endpoint was change from baseline to Week 3 in endurance time, measured by constant work rate cycle ergometry testing at 75% peak incremental work rate. Changes from baseline in intensity of exertional dyspnea (Borg CR10 Scale^®^) and trough inspiratory capacity were secondary endpoints. Additional endpoints included changes from baseline in other spirometric, plethysmographic, and physical activity (assessed by objective accelerometer measurement) parameters. Efficacy endpoints were analyzed using an analysis of covariance model.

**Results:**

In total, 112 patients were randomized and treated (mean age 60.3 years; mean post-bronchodilator forced expiratory volume in 1 s 1.7 L [56.7% predicted]; mean endurance time 485.7 s). After 3 weeks, endurance time was significantly increased with aclidinium versus placebo (treatment difference 58.5 s; p < 0.05). At Week 3, aclidinium significantly reduced dyspnea intensity at isotime during exercise (treatment difference -0.63; p < 0.05) and improved trough inspiratory capacity (treatment difference 78 mL; p < 0.05) versus placebo. Significant improvements in spirometric, plethysmographic, and some physical activity parameters were observed with aclidinium versus placebo.

**Conclusions:**

These results suggest that aclidinium significantly improves exercise endurance, exertional dyspnea, hyperinflation, and physical activity in patients with COPD.

**Trial registration:**

ClinicalTrials.gov identifier: NCT01471171; URL: http://www.clinicaltrials.gov.

**Electronic supplementary material:**

The online version of this article (doi:10.1186/1471-2466-14-209) contains supplementary material, which is available to authorized users.

## Background

Exercise limitation, driven predominantly by activity-related dyspnea, is an important feature of chronic obstructive pulmonary disease (COPD) that compromises daily living activities, leads to physical deconditioning, and contributes to a reduced perceived quality of life [1–6]. Lung hyperinflation is thought to represent a mechanical link between the characteristic expiratory airflow impairment, dyspnea, and exercise capacity [5, 6].

In patients with COPD, expiratory flow limitation and reduced lung elastic recoil result in air trapping and an increased end-expiratory lung volume (EELV) compared with healthy individuals [5–8]. During physical activity, acute increases in EELV (dynamic hyperinflation) occur and dyspnea is exacerbated, which can lead to avoidance of activity [5–8]. Alleviating hyperinflation and improving dyspnea, exercise tolerance, and levels of physical activity are important therapeutic goals in the management of COPD [9].

While there is a large body of clinical evidence regarding the effect of long-acting bronchodilators, including long-acting muscarinic antagonists (LAMAs), on exercise endurance [10–13], little is known about the translation of such improvements into changes in everyday physical activity. Aclidinium bromide is a novel LAMA recently approved, at a dose of 400 μg twice daily (BID), as a treatment for COPD [14, 15]. In a previous study, aclidinium 200 μg once daily (QD) significantly improved exercise endurance time, exertional dyspnea, and static hyperinflation versus placebo in patients with COPD [16]. Here we report results from a randomized, double-blind Phase IIIb crossover study to evaluate the effect of aclidinium 400 μg BID on cycling exercise endurance, exertional dyspnea, and lung hyperinflation in patients with COPD. A thorough profiling of lung volumes and objective measures of daily physical activity are also included.

## Methods

### Study design and treatment

**Figure 1:**
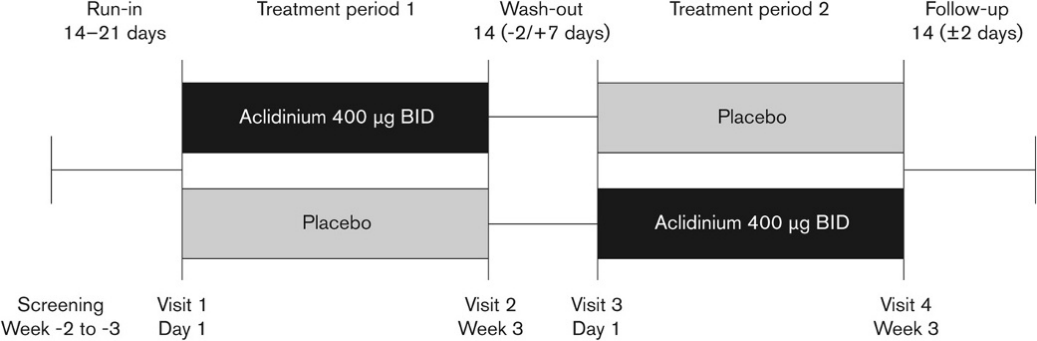
**Study design.** Symptom-limited incremental cycle exercise testing was performed at screening to determine maximum work rate. Constant work rate exercise testing at 75% of symptom-limited maximum work rate was conducted pre-dose at Visits 1 and 3 (baseline), and at 3 h post-dose at Visits 2 and 4. Spirometry was performed pre-dose at Visits 1 and 3 (baseline) and at Visits 2 and 4; plethysmography was performed pre-dose at Visits 1 and 3 (baseline), and at 2 h post-dose at Visits 2 and 4. BID, twice daily.

This Phase IIIb randomized, double-blind, crossover study was conducted between November 2011 and June 2012 at 14 sites in Germany, Spain, and the UK (clinicaltrials.gov identifier: NCT01471171). Following screening and a 2- to 3-week run-in period (including a visit 1 week before Visit 1 to familiarize patients with study procedures), patients were randomized (1:1; Visit 1) to aclidinium 400 μg (metered dose; equivalent to aclidinium 322 μg delivered dose) BID or placebo, each for 3 weeks in one of two sequences, with a 2-week washout between treatments (Figure 1).

Aclidinium and matched placebo were administered BID (09:00 ± 1 h and 21:00 ± 1 h) via a dry powder inhaler (Genuair^®^/Pressair^®^^a^). Randomization was performed according to unique patient identification numbers and a computer-generated random allocation sequence; patients and investigators were blinded to treatment allocation throughout the study.

Treatment with other long-acting bronchodilators was not permitted during the study. Patients were required to have discontinued treatment with LAMAs at least 7 days prior to the screening visit. Treatment with long-acting β_2_-agonists (LABAs) must have been discontinued at least 48 h prior to screening for BID LABAs and at least 7 days prior to screening for QD LABAs. Relief medication (salbutamol pressurized metered dose inhaler 100 μg/puff) was provided for symptom control as needed (except ≤6 h prior to lung function testing at each visit). The following maintenance therapies were also permitted if their use had been stable for ≥4 weeks prior to study entry: oral sustained-release theophylline, inhaled corticosteroids, and oral or parenteral corticosteroids (prednisone ≤10 mg per day or ≤20 mg every other day). Corticosteroid use was required to be suspended for ≥6 h prior to lung function testing at any visit.

### Study population

Patients aged ≥40 years with stable COPD and moderate-to-severe airflow limitation (post-bronchodilator forced expiratory volume in 1 s [FEV_1_] ≥30% and <80% of the predicted value, and FEV_1_/forced vital capacity [FVC] <70% at screening [9]), who were current or former cigarette smokers (smoking history of ≥10 pack-years) and had a functional residual capacity (FRC) of ≥120% of the predicted value at screening were eligible. Severity of dyspnea was not a specific inclusion criterion in the study.

Patients with a history of asthma, or any other clinically significant respiratory, cardiovascular or other systemic condition that may have contributed to dyspnea and exercise limitation, were not eligible to participate. Additional exclusion criteria included a respiratory tract infection or COPD exacerbation within 6 weeks prior to screening (within 3 months if exacerbation resulted in hospitalization), a requirement for long-term oxygen therapy (≥15 h/day), an inability to use the study inhaler, and contraindications for either the use of anticholinergic drugs or cardiopulmonary exercise testing [17]. Patients who, in the investigator’s opinion, may have needed to start a pulmonary rehabilitation program during the study and/or patients who started/finished a pulmonary rehabilitation program within 3 months prior to the screening visit were also excluded.

A symptom-limited incremental cycle exercise test was performed at screening to determine the maximum work rate that patients were able to maintain for ≥30 s. Patients who cycled <2 min or >20 min during exercise testing at 75% of maximum work rate during the run-in visit or Visit 1 (pre-randomization) were not permitted to continue in the study.

Patients could be discontinued from the study at any time at their own request or in the event of ineligibility, non-compliance, lack of efficacy, loss to follow-up (non-attendance), safety concerns (including moderate or severe COPD exacerbation), or any other reason at the investigator’s discretion.

The study was approved by the Independent Ethics Committees at each site (see Additional file 1) and was conducted in accordance with the Declaration of Helsinki, International Conference on Harmonisation, and Good Clinical Practice. Patients provided written informed consent before participating in any study procedures.

### Study assessments and endpoints

Constant work rate cycle ergometry testing at 75% maximum work rate was conducted pre-dose at Day 1 (baseline) of each treatment period (Visits 1 and 3), and at 3 h post-dose at Week 3 of each period (Visits 2 and 4). This consisted of 3 min rest followed by 3 min unloaded cycling before the constant phase, in which work rate was increased to 75% of maximum work rate and patients were encouraged to maintain a pedaling rate of 50–70 rotations per min until symptom limitation. Change from baseline in exercise endurance time (time from the start of the loaded test to the point of symptom limitation) to Week 3 was the primary endpoint.

Before exercise, every 2 min during, and at the end of exercise, patients rated the intensity of their dyspnea using the Borg CR10 Scale^®^ (perceived exertion, range: 0 = ‘nothing at all’ to 10 = ‘extremely strong/maximal’). If patients considered the intensity of their dyspnea to be stronger than ‘extremely strong/maximal’, they could use a larger numerical value (‘absolute maximum’). During exercise, inspiratory capacity (IC) was measured spirometrically at the same time points as a marker of dynamic hyperinflation. Change from baseline to Week 3 in intensity of dyspnea at isotime (duration of the shortest exercise test during Visits 1, 2, 3, and 4) was a secondary study endpoint. Changes from baseline to Week 3 in IC before exercise, every 2 min during exercise, at isotime, and at the end of exercise were additional endpoints. EELV at rest, at isotime, and at the end of exercise was calculated post-hoc by subtracting IC at each time point from mean total lung capacity (TLC) at 2 h post-dose. Oxygen saturation (SpO_2_) during exercise was assessed using a pulse oximeter.

Change from baseline in resting IC at Week 3 was also a secondary endpoint. Resting IC was assessed spirometrically pre-dose (trough) at each visit, according to American Thoracic Society and European Respiratory Society guidelines [18, 19]. Changes from baseline at Week 3 in trough FEV_1_ and FVC were also measured. All spirometry data were transferred for centralized reading and underwent a two-step quality-control process. Resting FRC, residual volume (RV), TLC, and specific airway conductance (sGaw) were assessed by whole-body plethysmography, performed pre-dose on Day 1 of each treatment period, and pre-dose and at 2 h post-dose at Week 3 of each period.

To assess physical activity, patients wore an armband device (SenseWear Pro3^®^, BodyMedia Inc., Pittsburgh, PA, USA) day and night (excluding time spent for personal hygiene purposes) for 7 days prior to the start of, and during the last week of, each treatment period. Changes from baseline in steps per day, minutes of at least moderate activity (>3 metabolic equivalents) per day, mean daily active energy expenditure >3 metabolic equivalents (kcal per day), and mean physical activity level (PAL; total energy expenditure divided by resting energy expenditure) [20] at Week 3 were additional efficacy endpoints. Baseline PAL values were also used to categorize patients as ‘extremely inactive’ (PAL <1.40), ‘sedentary’ (PAL 1.40–1.69), and ‘at least moderately active’ (PAL >1.70) [20]. The frequency of nocturnal awakenings due to COPD symptoms and relief medication use were also recorded daily using patient diaries.

Safety assessments included monitoring of adverse events (AEs) throughout the study, laboratory assessments at screening and Visit 4, and physical examination, blood pressure measurement, and 12-lead electrocardiogram at screening and at Visits 1 and 4.

### Statistical analysis

A population of 84 patients was estimated to provide >85% power to detect a treatment group difference of 120 s for the primary endpoint. The study, therefore, planned to screen 170 patients and to randomize 110 patients, allowing for a 35% screening failure rate and a 20% drop-out rate.

Analyses of efficacy variables were performed on the intent-to-treat (ITT) population (all randomized patients who took ≥1 dose of study drug and had ≥1 baseline and post-dose assessment for the primary endpoint in ≥1 of the two treatment periods). Efficacy endpoints were analyzed using an analysis of covariance model with treatment and period as fixed effects, patients as a random effect, and baseline values as covariates. Least squares means and 95% confidence intervals (CI) were calculated for treatment group differences (∆). Adjustment for multiplicity was performed using a step-down approach: if the comparison between aclidinium and placebo for the primary endpoint (exercise endurance time) was significant at the 5% level, then the secondary endpoints were tested in turn (trough IC first, intensity of dyspnea at isotime second). EELV at baseline and at Week 3 was analyzed descriptively. Only patients with ≥5 days with ≥22 h valid data were included in the analysis of physical activity parameters [20].

Post-hoc analyses were performed to investigate the relationship between changes from baseline in endurance time and physical activity, and between changes from baseline in endurance time and lung volumes (Pearson Correlation Coefficients). In addition, changes from baseline in endurance time and trough IC at Week 3, and physical activity endpoints over the last week of treatment, were assessed according to baseline PAL. Analyses were performed using an analysis of covariance model with treatment and period as fixed effects, patients as a random effect, and baseline values as covariates.

Patient demographic and baseline characteristics, and safety outcomes, were summarized descriptively based on the safety population (all randomized patients who took ≥1 dose of study drug).

## Results

### Study population

**Figure 2:**
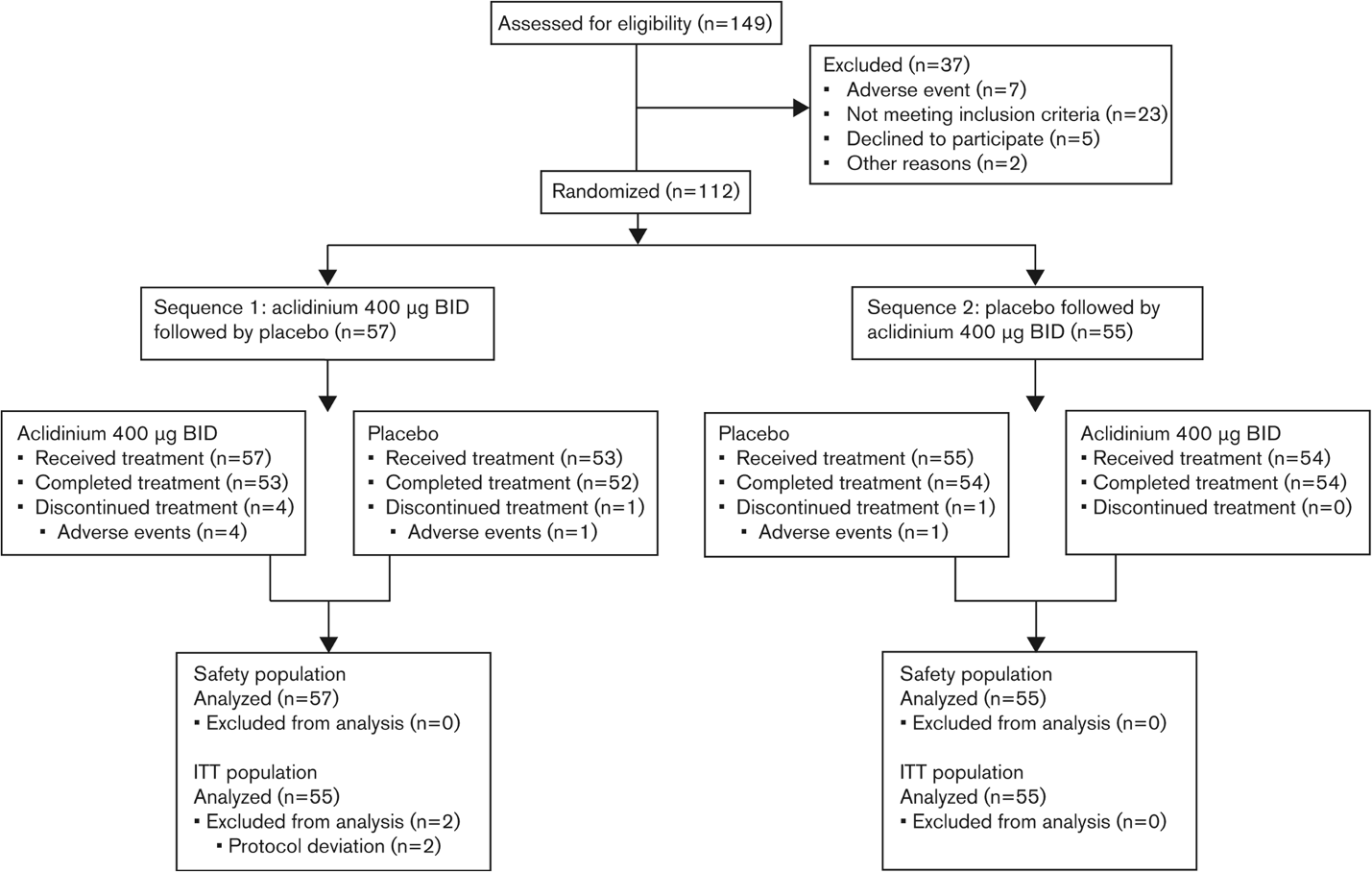
**Patient disposition.** BID, twice daily; ITT, intent-to-treat.

Of 149 patients screened, 112 were randomized and treated (safety analysis population) and 110 were included in the ITT population (Figure 2). The study was completed by 106 patients; 6 patients discontinued due to AEs.

Patient demographics and baseline characteristics are summarized in Table 1. Details of prior COPD medication use are shown in Additional file 2. Pre-dose at Visit 1, 49 patients (44.5%) in the ITT population developed dynamic hyperinflation during exercise (defined as a >150 mL decrease from rest in IC [21]).

**Table 1:** **Patient demographics and baseline characteristics (safety population)**

Characteristic	Patients (N = 112)
Age (years), mean (SD)	60.3 (8.1)
Gender (male), n (%)	76 (67.9)
Race (Caucasian/white), n (%)	112 (100.0)
Current smoker, n (%)	74 (66.1)
Smoking history (pack-years), mean (SD)	48.0 (25.0)
COPD duration (years), mean (SD)	8.8 (6.3)
Severity of airflow limitation,^a^ n (%)	
Moderate	80 (71.4)
Severe	32 (28.6)
Post-bronchodilator FEV_1_ (L) at screening	
Mean (SD)	1.7 (0.5)
% predicted, mean (SD)	56.7 (11.6)
FRC at screening (L)	
Mean (SD)	5.0 (1.0)
% predicted, mean (SD)	152.3 (26.0)
Exercise endurance time at baseline^b^ (s), mean (SD)	485.7 (234.4)
Intensity of dyspnea at isotime at baseline^b^ (Borg CR-10 scale), mean (SD)	5.7 (2.6)
Lung function variables at baseline,^b^ mean (SD)	
FEV_1_ (L)	1.5 (0.5)
% predicted FEV_1_	49.7 (14.7)
FVC (L)	3.3 (0.9)
% predicted FVC	88.3 (18.2)
IC (L)	2.2 (0.5)
Whole-body plethysmographic variables at baseline,^b^ mean (SD)	
FRC (L)	5.0 (1.1)
% predicted FRC	151.3 (28.0)
RV (L)	4.0 (1.0)
% predicted RV	179.4 (40.5)
TLC (L)	7.3 (1.3)
% predicted TLC	116.9 (15.6)
sGaw (s^-1^kPa^-1^)	0.5 (0.3)
Physical activity variables at baseline,^c^ mean (SD)	
Step count (steps per day)	7030.0 (3718.3)
Duration of activity of at least moderate intensity^d^ (min/day)	92.3 (70.8)
Physical activity level^e^	1.514 (0.216)
Daily active energy expenditure >3 metabolic equivalents (kcal/day)	451.1 (372.6)

^a^GOLD Stage 2 (moderate): FEV_1_/FVC <0.70, and post-bronchodilator FEV_1_ ≥50% and <80% predicted; GOLD Stage 3 (severe): FEV_1_/FVC <0.70, and post-bronchodilator FEV_1_ ≥30% and <50% predicted.

^b^Pre-dose at Visit 1.

^c^Mean data for 7-day period prior to Visit 1 (patients who had ≥5 days with ≥22 h of valid data; n = 92).

^d^Any physical activity >3 metabolic equivalents.

^e^Ratio calculated as the total daily energy expenditure divided by the whole of the night sleeping energy expenditure.

COPD, chronic obstructive pulmonary disease; FEV_1_, forced expiratory volume in 1 s; FRC, functional residual capacity; FVC, forced vital capacity; GOLD, Global initiative for chronic Obstructive Lung Disease; IC, inspiratory capacity; RV, residual volume; SD, standard deviation; sGaw, specific airway conductance; TLC, total lung capacity.

### Efficacy endpoints

#### Exercise endurance, exertional dyspnea, and dynamic hyperinflation

**Figure 3:**
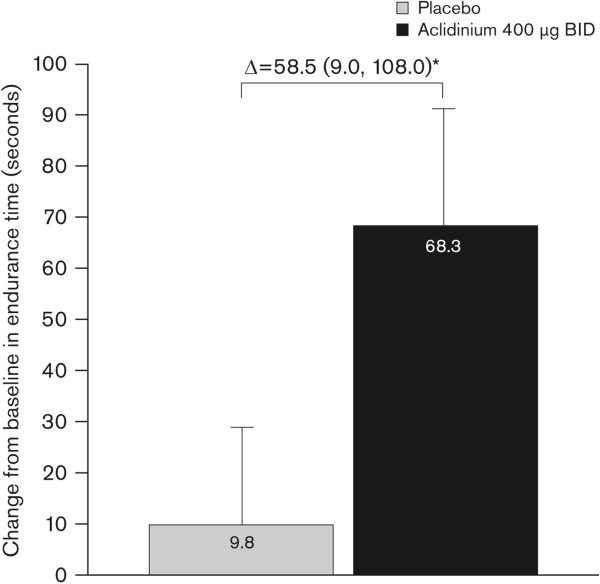
**Change from baseline in exercise endurance time at Week 3.** Change from baseline in exercise endurance time during constant work rate cycle ergometry to symptom limitation at 75% of the maximum work rate was assessed at Week 3 (ITT population). Data reported as least squares means change from baseline (analysis of covariance) + standard error; Δ = least squares means difference (95% confidence interval). ^*^p < 0.05 versus placebo. BID, twice daily; ITT, intent-to-treat.

After 3 weeks of treatment, change from baseline in exercise endurance time was significantly greater with aclidinium compared with placebo (Δ = 58.5 s; 95% CI 9.0, 108.0; p < 0.05; Figure 3). The percentage change from baseline was 9.3% with placebo and 18.2% with aclidinium at Week 3.

**Figure 4:**
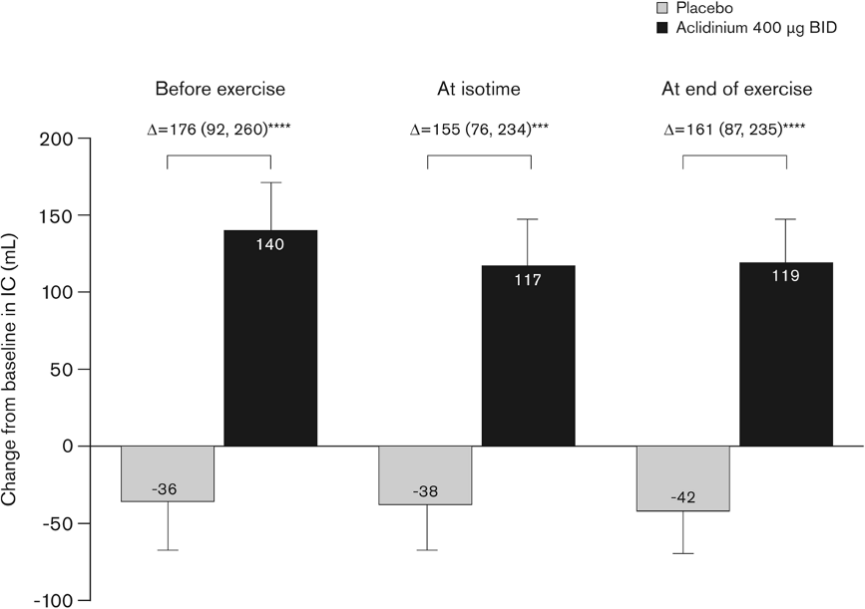
**Change from baseline in dynamic IC at end of exercise at Week 3.** Change from baseline in dynamic IC measured before exercise, at isotime, and at end of exercise was assessed at Week 3 (ITT population). Data reported as least squares means change from baseline (analysis of covariance) + standard error; Δ = least squares means difference (95% confidence intervals). ^***^p < 0.001, ^****^p < 0.0001 versus placebo. BID, twice daily; IC, inspiratory capacity; ITT, intent-to-treat.

**Figure 5:**
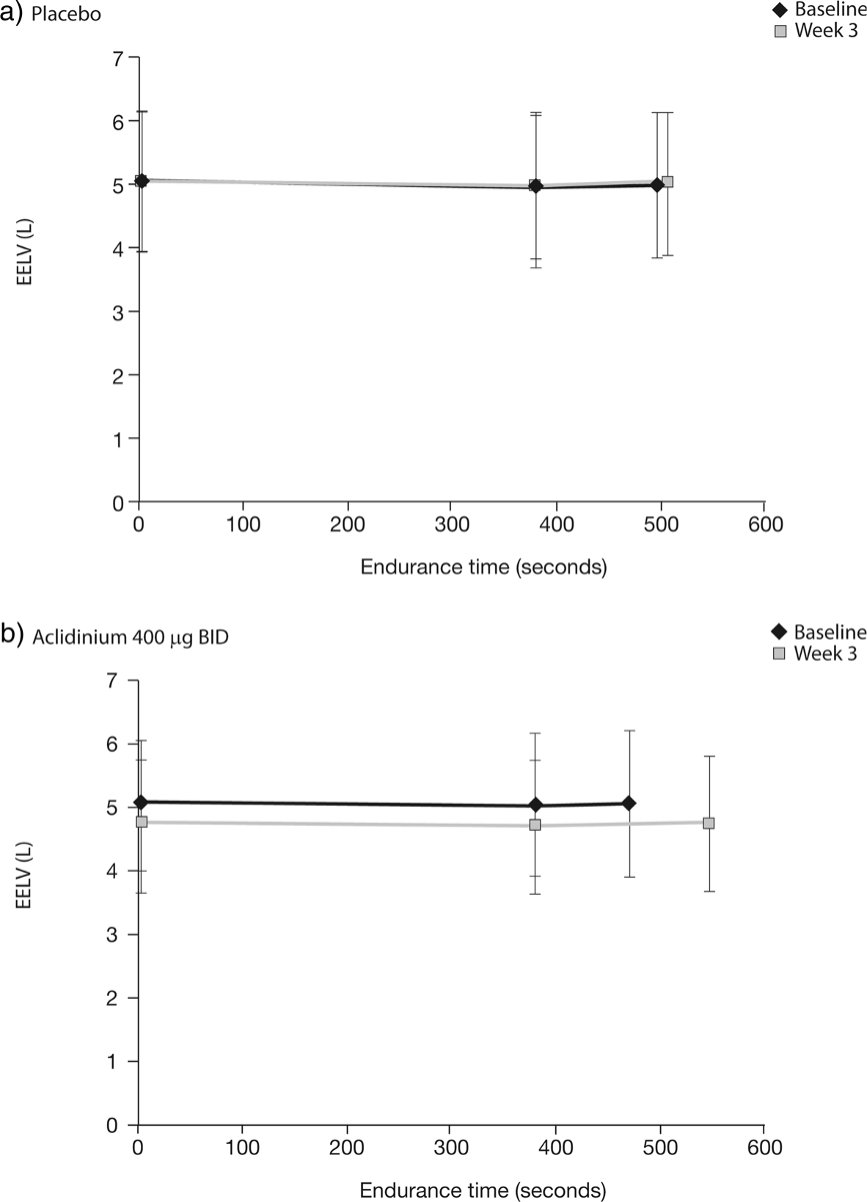
**EELV during exercise at baseline and at Week 3.** EELV during constant work rate cycle ergometry in patients receiving **a)** placebo, or **b)** aclidinium 400 μg BID. EELV was assessed at rest, at isotime, and at end of exercise at baseline and at Week 3 (ITT population). Data are descriptive and reported as mean ± standard deviation. EELV was assessed as IC subtracted from mean TLC 2 h post-dose. Mean isotime was calculated as 386 seconds. BID, twice daily; EELV, end-expiratory lung volumes; IC, inspiratory capacity; ITT, intent-to-treat; TLC, total lung capacity.

Compared with placebo, aclidinium also significantly reduced the intensity of dyspnea at isotime from baseline to Week 3 (Δ = -0.63; 95% CI -1.11, -0.14; p < 0.05) and significantly improved dynamic IC from baseline at Week 3 when measured before exercise, at isotime, and at the end of exercise (Figure 4). With aclidinium treatment, improvements from baseline in IC were seen for every 2-min interval during the first 10 min of exercise at Week 3 (range: 79 mL to 132 mL; Additional file 3). In contrast, IC decreased compared with baseline for each corresponding period in the placebo group (range -16 mL to -110 mL; Additional file 3). Aclidinium significantly improved IC compared with placebo at each interval from 0 to 8 min; there was no significant difference at 8–10 min. Consistent with improvements in dynamic IC, EELV at Week 3 was also reduced compared with baseline during exercise with aclidinium 400 μg BID (Figure 5). Reductions in EELV were observed at rest, at isotime, and at the end of exercise. In contrast, there was no change from baseline in EELV at Week 3 with placebo.

Throughout the study, only two patients did not reach 85% SpO_2_ during exercise, suggesting that oxygen desaturation was unlikely to be a major contributor to exercise limitation in these patients.

#### Static lung function and hyperinflation

After 3 weeks, aclidinium significantly increased trough IC from baseline versus placebo (Δ = 78 mL; 95% CI 10, 145; p < 0.05; Table 2). Other parameters of static lung function were also significantly increased from baseline with aclidinium versus placebo (Table 2).

**Table 2:** **Changes from baseline in lung function and body plethysmography parameters at Week 3 (ITT population)**

Parameter	Placebo (N = 108)	Aclidinium 400 μg BID (N = 109)	Treatment difference vs placebo (95% CI)
Pre-dose (trough) IC (mL)	20 (25)	98 (24)	78 (10, 145)^*^
Pre-dose (trough) FEV_1_ (mL)	-25 (21)	108 (21)	132 (74, 191)^****^
Pre-dose (trough) FVC (mL)	-46 (31)	198 (30)	243 (157, 329)^****^
FRC (mL)			
Pre-dose (trough)	15 (39)	-182 (39)	-197 (-321, -72)^**^
Post-dose	-130 (46)	-449 (46)	-318 (-448, -189)^****^
RV (mL)			
Pre-dose (trough)	15 (60)	-222 (60)	-238 (-396, -79)^**^
Post-dose	-81 (57)	-523 (57)	-443 (-599, -286)^****^
TLC (mL)			
Pre-dose (trough)	-6 (41)	-82 (41)	-76 (-201, 49)
Post-dose	-49 (41)	-199 (41)	-150 (-262, -37)^**^
sGaw (s^-1^kPa^-1^)			
Pre-dose (trough)	0.002 (0.020)	0.096 (0.020)	0.094 (0.038, 0.150)^**^
Post-dose	0.054 (0.027)	0.297 (0.026)	0.243 (0.182, 0.303)^****^

Data reported as least squares means (standard error) change from baseline (analysis of covariance).

^*^p < 0.05, ^**^p < 0.01, ^****^p < 0.0001 versus placebo.

BID, twice daily; CI, confidence interval; FEV_1_, forced expiratory volume in 1 s; FRC, functional residual capacity; FVC, forced vital capacity; IC, inspiratory capacity; ITT, intent-to-treat; RV, residual volume; sGaw, specific airway conductance; TLC, total lung capacity.

Mean trough FRC, RV, and sGaw were significantly improved from baseline with aclidinium versus placebo at Week 3 (Table 2). The mean change from baseline in trough IC:TLC ratio was 0.016 with aclidinium and 0.002 with placebo (Δ = 0.014; p < 0.01). At Week 3, significant improvements from baseline with aclidinium versus placebo were observed for mean post-dose FRC, RV, TLC, and sGaw (Table 2).

When assessed across the placebo and aclidinium treatment periods, there was a weak negative correlation between changes from baseline in endurance time and post-dose RV (r = -0.159; p < 0.05), post-dose FRC (r = -0.175; p = 0.01), and trough FRC (r = -0.144; p < 0.05), and a weak positive correlation between changes from baseline in endurance time and IC at rest (r = 0.231; p < 0.001). There was no significant correlation between changes in endurance time and trough RV and trough IC. In the aclidinium treatment period, correlations between lung volumes and endurance time were weak and did not reach statistical significance.

#### Physical activity and symptoms

Changes from baseline in physical activity, as measured by duration of activity of at least moderate intensity and daily active energy expenditure, were significantly increased with aclidinium versus placebo at Week 3 (Table 3). Numeric increases in step count and physical activity level were also observed with aclidinium versus placebo but did not reach significance (Table 3).

**Table 3:** **Changes from baseline in physical activity parameters at Week 3 (ITT population)**

Parameter	Placebo (n = 83 ^a^)	Aclidinium 400 μg BID (n = 85 ^a^)	Treatment difference vs placebo (95% CI)
Step count (steps per day)	-163.2 (226.1)	295.8 (223.7)	459.0 (-61.8, 979.8)
Duration of activity of at least moderate intensity^b^ (min/day)	-5.9 (4.1)	4.2 (4.1)	10.1 (2.0, 18.2)^*^
Physical activity level^c^	-0.006 (0.013)	0.018 (0.013)^d^	0.024 (-0.003, 0.051)
Daily active energy expenditure >3 metabolic equivalents (kcal/day)	-32.7 (21.0)	21.9 (20.8)	54.5 (13.3, 95.8)^*^

Data reported as least squares means (standard error) change from baseline (analysis of covariance).

^*^p < 0.05 versus placebo.

^a^n = number of patients who had ≥5 days with ≥22 h of valid data and were included in the analyses.

^b^Any physical activity >3 metabolic equivalents.

^c^Ratio calculated as the total daily energy expenditure divided by the whole of the night sleeping energy expenditure.

^d^n = 84.

BID, twice daily; CI, confidence interval; ITT, intent-to-treat.

When assessed across both treatments, there was no significant correlation between changes from baseline in step count or moderate activity and changes from baseline in endurance time (Table 4). Improvements in physical activity parameters were related, and changes from baseline in endurance time were negatively correlated with changes in dyspnea at isotime (Table 4). As shown in Additional file 4, similar results were observed during the aclidinium period only.

**Table 4:** **Relationship between physical activity, endurance time, and exertional dyspnea**

		Observations ^a^	Correlation	P-value
Change from baseline in daily step count^b^	Change from baseline in endurance time (s)	168	0.061	0.433
	Change from baseline in duration of at least moderate activity (min)^b^	168	0.640	<0.0001
Change from baseline in duration of at least moderate activity (min/day)^b^	Change from baseline in endurance time (s)	168	-0.015	0.845
Change from baseline in endurance time (s)	Change from baseline in exertional dyspnea at isotime	216	-0.535	<0.0001

Pearson correlation for change from baseline in mean daily step count, mean duration of at least moderate activity (>3 metabolic equivalents), endurance time during constant work rate cycle ergometry to symptom limitation at 75% of the maximum work rate, and exertional dyspnea at isotime across the study (aclidinium and placebo treatment periods; ITT population).

^a^Number of observations used to calculate the correlation.

^b^Patients who had ≥5 days with ≥22 h of valid data.

ITT, intent-to-treat.

Over 3 weeks there was a significant increase in relief medication-free days with aclidinium versus placebo (6.3% vs 20.9%; Δ = 14.6%; p < 0.0001). Reductions in number of nocturnal awakenings due to COPD symptoms were small and there was no significant difference between treatments (p = 0.46).

#### Treatment effect by baseline PAL

**Figure 6:**
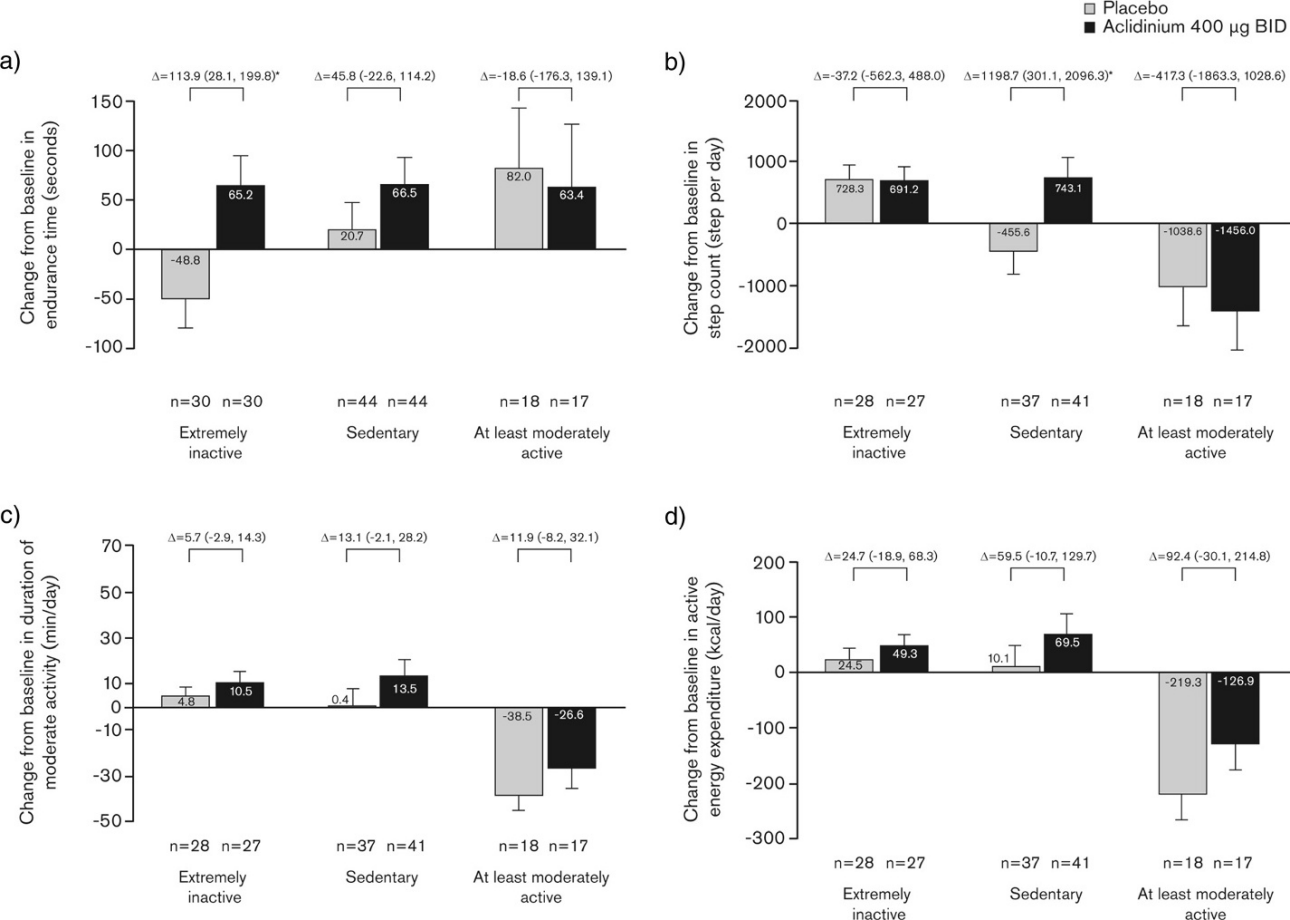
**Changes from baseline in exercise endurance and physical activity at Week 3, by baseline physical activity.** Changes from baseline in **a)** exercise endurance time during constant work rate cycle ergometry to symptom limitation at 75% of the maximum work rate, **b)** daily step count, **c)** duration of moderate activity >3 metabolic equivalents, and **d)** energy expenditure at Week 3 were assessed according to baseline physical activity levels (inactive, sedentary, or at least moderately active; ITT population). Data reported as least squares means change from baseline (analysis of covariance) + standard error; Δ = least squares means difference (95% confidence intervals). n = number of patients in each subgroup included in the analyses. ^*^p < 0.05 versus placebo. BID, twice daily; ITT, intent-to-treat.

Post-hoc analyses assessed change from baseline in endurance time at Week 3 in patients categorized as ‘extremely inactive’, ‘sedentary’, and ‘at least moderately active’ at baseline. Improvements in endurance time with aclidinium compared with placebo were greatest in patients who were categorized as extremely inactive at baseline (Figure 6a). While there were also improvements in endurance time with aclidinium versus placebo in patients who were sedentary at baseline, these improvements were not statistically significant.

When physical activity parameters were assessed, improvements with aclidinium versus placebo were generally greatest in patients who were categorized as sedentary at baseline (Figure 6). In these patients, the improvement in daily step count was significantly greater with aclidinium versus placebo (Figure 6).

### Safety

The incidence of AEs was higher with aclidinium (44.1%) than with placebo (30.6%). The most common AEs were nasopharyngitis, headache, and abnormal (bitter) product taste; each reported by a higher proportion of patients receiving aclidinium (6.3%, 4.5%, and 3.6%, respectively) than placebo (3.7%, 2.8%, and 1.9%, respectively). There were few discontinuations due to AEs following aclidinium and placebo (3.6% and 1.9%, respectively). No serious AEs occurred during treatment with aclidinium. There were no clinically relevant changes in hematology or biochemistry parameters, or in other safety variables.

## Discussion

The results of this study show that after 3 weeks, aclidinium 400 μg BID significantly reduced the intensity of exertional dyspnea and improved cycling exercise endurance compared with placebo. As lung hyperinflation is believed to be an important factor contributing to reduced exercise capacity in COPD [5–8], we believe the main driver of increased exercise endurance was the consistent effect of aclidinium treatment on lung volumes, as reflected by significant improvements in static IC, FRC, RV, and TLC, as well as dynamic IC during exercise.

The improvement in endurance time observed with aclidinium was similar to that observed previously with tiotropium 18 μg QD (67 s) [10], but lower than that seen with glycopyrrolate 50 μg QD (89 s) [13]. Improvements, versus placebo, in IC at isotime (a marker of dynamic hyperinflation) were also similar between aclidinium 400 μg BID (155 mL), tiotropium 18 μg QD (180–190 mL) [10, 22], and glycopyrrolate 50 μg QD (200 mL) [13]. Furthermore, the improvement in static lung function (IC at rest) with aclidinium (176 mL) was also comparable to that provided by other LAMAs (180–220 mL) [10, 13, 22].

It has been proposed that a minimal clinically important difference for exercise endurance time is in the range 46–105 s [23, 24]; however, these estimates are based on few studies and do not take into account the large variation in, for example, interventions, study methodology/duration, patient characteristics and disease severity, and exercise testing protocols. Furthermore, it is unclear if a change observed with a pulmonary rehabilitation program involving regular training units is comparable to what can be achieved with bronchodilators. Nevertheless, most published improvements in cycling endurance with bronchodilators are in line with the proposed range. We therefore believe that the improvements seen with aclidinium fit well with the published data and indicate a meaningful response.

In the present study, there was a correlation between improvements in endurance time and reduced exertional dyspnea, overall and in the aclidinium treatment period only. Overall, improvements in endurance time were also correlated with reduced post-dose RV and FRC and increased IC at rest. There was no significant correlation between changes from baseline in static lung volumes and endurance time when assessed in the aclidinium treatment period only. To the best of our knowledge, only one previous study has investigated the relationship between exercise endurance time and lung volumes following treatment with a LAMA in patients with COPD [10]. While a correlation between endurance time and exertional dyspnea and between endurance time and dynamic IC was observed following treatment with tiotropium 18 μg; the relationship between endurance time and static lung volumes was not reported [10]. Furthermore, patients were treated with tiotropium 18 μg for 6 weeks compared with the shorter 3-week treatment period with aclidinium in the present study.

Aclidinium treatment also significantly improved the duration of activity of moderate intensity and daily energy expenditure. It is difficult to speculate on the clinical relevance of the improvement in the duration of moderate physical activity observed with aclidinium (10 min vs placebo). Recently, a prospective cohort study reported the health benefits of 15 min moderate physical activity per day in terms of mortality reduction in the general population [25]. Furthermore, physical activity level has been found to be a strong predictor of mortality in patients with COPD [26, 27]. Further studies are needed to link improvements in physical activity in COPD to other patient-centered outcomes, and ideally, to also demonstrate health benefits in terms of reduced morbidity and mortality.

In the present study, there was no correlation between improved endurance time and increased physical activity in the whole study population and following treatment with aclidinium. This might be based on the observation that patients in this study who were extremely inactive at baseline had the greatest improvements in endurance time, whereas patients who were sedentary at baseline had the greatest improvements in physical activity. While the analyses reported here were exploratory, and in the case of the baseline PAL analyses, performed in small numbers of patients, they expand previous studies that suggest factors other than increased capacity are involved in driving patients with COPD to become more physically active.

Study limitations might include the short treatment duration, absence of assessments beyond 3 h post-dose to determine duration of effect, and the lack of an active comparator. Nevertheless, this study provides useful data to supplement findings from previous Phase III studies, demonstrating that aclidinium significantly improved lung function and symptoms versus placebo over 12 and 24 weeks and had comparable 24-h bronchodilatory efficacy to tiotropium over 6 weeks in patients with COPD [28–30]. Aclidinium has also been demonstrated to be safe and well tolerated in patients with COPD [29, 30], and the safety findings from this short study were consistent with those previously observed.

## Conclusions

Treatment with aclidinium 400 μg BID significantly improved exercise endurance and related exertional dyspnea, and lung hyperinflation compared with placebo over 3 weeks in patients with stable COPD and moderate-to-severe airflow limitation. Aclidinium also provided significant improvements in static lung function and lung volumes, and parameters of physical activity compared with placebo.

## Endnote

^a^Registered trademarks of AstraZeneca PLC, Barcelona, Spain; for use within the USA as Pressair^®^, and Genuair^®^ within all other licensed territories.

## Author’s information

Diana Jarreta Formerly of Almirall S.A., Barcelona, Spain.

Esther Garcia Gil Formerly of Almirall S.A., Barcelona, Spain.

## Electronic supplementary material

Additional file 1: **Institutions and Independent Ethics Committees.** Details of institutions and Independent Ethics Committees. (PDF 92 KB)

Additional file 2: **Prior COPD medication use by therapeutic category.** Number of patients using any pre-study medication for COPD. (PDF 73 KB)

Additional file 3: **Change from baseline in IC during exercise at Week 3.** Changes from baseline in inspiratory capacity every 2 min during exercise (assessed by constant work rate cycle ergometry). (PDF 36 KB)

Additional file 4: **Relationship between physical activity, endurance time, and exertional dyspnea.** Pearson correlation for change from baseline in mean daily step count, mean duration of at least moderate activity (>3 metabolic equivalents), endurance time during constant work rate cycle ergometry to symptom limitation at 75% of the maximum work rate, and exertional dyspnea at isotime across the study during the aclidinium treatment period only. (PDF 71 KB)

## Abbreviations

AE: Adverse event
BID: Twice daily
CI: Confidence interval
COPD: Chronic obstructive pulmonary disease
EELV: End-expiratory lung volume
FEV_1_: Forced expiratory volume in 1 s
FRC: Functional residual capacity
FVC: Forced vital capacity
IC: Inspiratory capacity
ITT: Intent-to-treat
LABA: Long-acting β2-agonist
LAMA: Long-acting muscarinic antagonist
PAL: Physical activity level
QD: Once daily
RV: Residual volume
sGaw: specific airway conductance
SpO_2_: Oxygen saturation
TLC: Total lung capacity
Δ: Treatment group difference.
