# Supplementary material for: Aclidinium improves exercise endurance, dyspnea, lung hyperinflation, and physical activity in patients with COPD: a randomized, placebo-controlled, crossover trial
Source: BMC Pulm Med. 2014 Dec 23;14:209. doi: 10.1186/1471-2466-14-209 (PMC4364572; doi:10.1186/1471-2466-14-209)
Supplement: Supplementary file 1 — Additional file 1: Institutions and Independent Ethics Committees. Details of institutions and Independent Ethics Committees. (PDF 92 KB) [file 12890_2014_644_MOESM1_ESM.pdf]

Table. Institutions and Independent Ethics Committees.

Institution	Name, address and chairperson of local IEC
Germany	
INSAF GmbH<br>Biebricher Allee 34<br>65187 Wiesbaden	Ethikkommission der Landesärztekammer Hessen<br>Im Vogelsgesang 3<br>60488 Frankfurt am Main<br>Chairperson: Professor Dr S Harder
Praxis Gesine Groth, Dr Med<br>Erdkampsweg 49<br>Hamburg	Ethikkommission der Ärztekammer Hamburg<br>Humboldtstr. 67a<br>22083 Hamburg<br>Chairperson: Professor Dr T Weber
Klin. Forschung Berlin Mitte GmbH<br>Georgenstraße 24<br>10117 Berlin	Ethikkommission des Landes Berlin<br>Landesamt für Gesundheit und<br>Soziales<br>Fehrbelliner Platz 1<br>10707 Berlin<br>Chairperson: Professor Dr Fülle
PAREXEL International GmbH<br>Spandauer Damm 130 Haus 31<br>14050 Berlin	Ethikkommission des Landes Berlin<br>Landesamt für Gesundheit und<br>Soziales<br>Fehrbelliner Platz 1<br>10707 Berlin<br>Chairperson: Professor Dr Fülle
Hamburger Institut für Therapieforschung GmbH<br>Colonnaden 72	Ethikkommission der Ärztekammer Hamburg<br>Humboldtstr. 67a

20354 Hamburg	22083 Hamburg<br>Chairperson: Professor Dr T Weber
KLB Gesundheitsforschung Lübeck GmbH<br>Pferdemarkt 6-8<br>23552 Lübeck	Ethikkommission der Ärztekammer Schleswig-Holstein<br>Bismarckallee 8-12<br>23795 Bad Segeberg<br>Chairperson: Professor Dr G Hintze
Fraunhofer-Institut für Toxikologie und Experimentelle Medizin ITEM<br>Nikolai-Fuchs-Straße 1a<br>30625 Hannover	Ethikkommission der Medizinischen Hochschule Hannover<br>Carl-Neuberg-Str. 1<br>30625 Hannover<br>Chairperson: Professor Dr HD Tröger
Charité Research Organisation<br>Charitéplatz 1<br>10117 Berlin	Ethikkommission des Landes Berlin<br>Landesamt für Gesundheit<br>und Soziales<br>Fehrbelliner Platz 1<br>10707 Berlin<br>Chairperson: Professor Dr Fülle
Pneumologisches Forschungsinstitut am KH Großhansdorf<br>Wöhrendamm 80<br>22927 Großhansdorf	Ethikkommission der Ärztekammer Schleswig-Holstein<br>Bismarckallee 8-12<br>23795 Bad Segeberg<br>Chairperson: Professor Dr G Hintze

IKF Pneumologie<br>Stresemannallee 3<br>Schaumainkai 101-103<br>60596 Frankfurt	Ethikkommission der Landesärztekammer Hessen<br>Im Vogelsgesang 3<br>60488 Frankfurt am Main<br>Chairperson: Professor Dr S Harder
Spain	
Clínica Mediterránea de Neurociencias<br>Neumología Vía Parque<br>s/n Camino Viejo Alicante-Elche<br>03114 Alicante	CEIC Clínica Mediterránea de Neurociencias<br>Via Parque Alicante – Elche<br>s/n 03114 Partida de Bacarot Alicante<br>Chairperson: Not applicable
Hospital General Universitario Gregorio Marañón. Lab.<br>Pruebas Funcionales C/ Dr Esquerdo, 46<br>28007 Madrid	CEIC Área 1 - Hospital General Universitario Gregorio Marañón<br>Fundación para la Investigación Biomédica<br>Pabellón de Gobierno<br>Planta baja<br>C/ Dr Esquerdo, 46<br>28007<br>Madrid<br>Chairperson: Not applicable

Hospital del Mar.<br>Servicio de Neumología Passeig del Mar, 25-29<br>08003 Barcelona	CEIC Parc de Salut Mar Consorci Mar Parc de Salut de Barcelona<br>Parc de Recerca Biomèdica de Barcelona (dcho. 163.03)<br>C/ Doctor Aiguader, 88<br>1ª planta<br>08003<br>Barcelona<br>Chairperson: Not applicable
UK	
Queen Anne Street Medical Centre<br>18-22 Queen Anne Street<br>London<br>W1G 8HU	London - Fulham REC<br>Charing Cross Hospital<br>Fulham Palace Road<br>London<br>W6 8RF<br>Chairperson: Not applicable

IEC, Independent Ethics Committee.
