# Supplementary material for: Aclidinium improves exercise endurance, dyspnea, lung hyperinflation, and physical activity in patients with COPD: a randomized, placebo-controlled, crossover trial
Source: BMC Pulm Med. 2014 Dec 23;14:209. doi: 10.1186/1471-2466-14-209 (PMC4364572; doi:10.1186/1471-2466-14-209)
Supplement: Supplementary file 2 — Additional file 2: Prior COPD medication use by therapeutic category. Number of patients using any pre-study medication for COPD. (PDF 73 KB) [file 12890_2014_644_MOESM2_ESM.pdf]

Table. Prior COPD medication use according to therapeutic category. The proportion of patients using pre-study medication for COPD. Pre-study medication was defined as any medication taken within 15 days prior to informed consent up to the date of informed consent (safety population).

**COPD medication category, n (%)[a]**	**N=112**
Any COPD medication	100 (89.3)
SABA	84 (75.0)
LAMA	37 (33.0)
LABA	22 (19.6)
LABA + ICS	22 (19.6)
ICS	15 (13.4)
Other[b]	9 (8.0)
SABA + SAMA	6 (5.4)

[a]Patients were counted separately in each of the medication categories.

[b]COPD medications used by ≤3 patients each. Includes SAMAs, xanthines, influenza vaccines, oral PDE4 inhibitors, oxygen and systemic corticosteroids.

COPD, chronic obstructive pulmonary disease; ICS, inhaled corticosteroid; LABA, long-acting β_2-agonist; LAMA, long-acting muscarinic antagonist; PDE4, phosphodiesterase-4; SABA, short-acting β_2-agonist; SAMA, short-acting muscarinic antagonist.
