# Supplementary material for: Aclidinium improves exercise endurance, dyspnea, lung hyperinflation, and physical activity in patients with COPD: a randomized, placebo-controlled, crossover trial
Source: BMC Pulm Med. 2014 Dec 23;14:209. doi: 10.1186/1471-2466-14-209 (PMC4364572; doi:10.1186/1471-2466-14-209)
Supplement: Supplementary file 3 — Additional file 3: Change from baseline in IC during exercise at Week 3. Changes from baseline in inspiratory capacity every 2 min during exercise (assessed by constant work rate cycle ergometry). (PDF 36 KB) [file 12890_2014_644_MOESM3_ESM.pdf]

Figure. **Change from baseline in IC during exercise at Week 3**. Change from baseline in IC every 2 min during exercise (constant work rate cycle ergometry; ITT population).

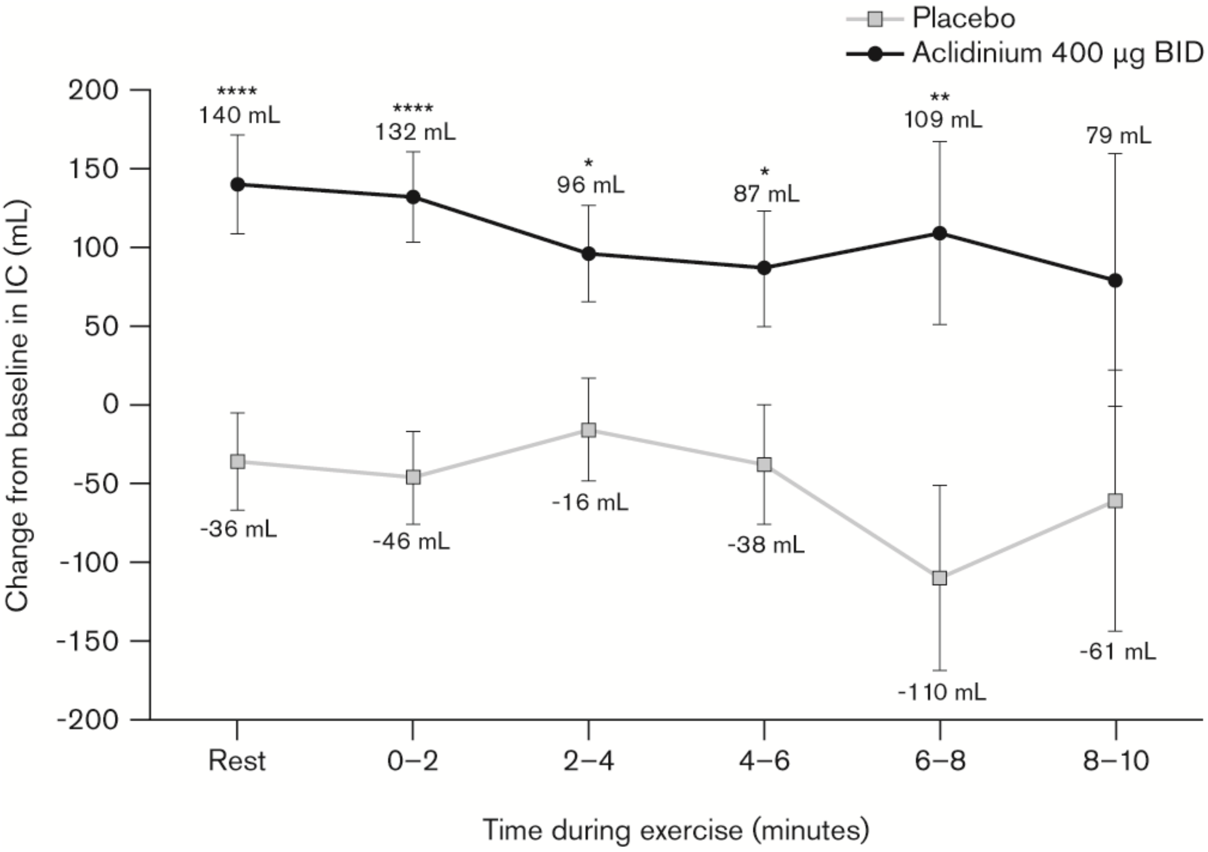

Data reported as least squares means change from baseline (analysis of covariance) + standard error; *p<0.05, **p<0.01, ****p<0.0001 versus placebo.

BID, twice daily; IC, inspiratory capacity; ITT, intent-to-treat.
