# Supplementary material for: Aclidinium improves exercise endurance, dyspnea, lung hyperinflation, and physical activity in patients with COPD: a randomized, placebo-controlled, crossover trial
Source: BMC Pulm Med. 2014 Dec 23;14:209. doi: 10.1186/1471-2466-14-209 (PMC4364572; doi:10.1186/1471-2466-14-209)
Supplement: Supplementary file 4 — Additional file 4: Relationship between physical activity, endurance time, and exertional dyspnea. Pearson correlation for change from baseline in mean daily step count, mean duration of at least moderate activity (>3 metabolic equivalents), endurance time during constant work rate cycle ergometry to symptom limitation at 75% of the maximum work rate, and exertional dyspnea at isotime across the study during the aclidinium treatment period only. (PDF 71 KB) [file 12890_2014_644_MOESM4_ESM.pdf]

Table. **Relationship between physical activity, endurance time, and exertional dyspnea.** Pearson correlation for change from baseline in mean daily step count, mean duration of at least moderate activity (>3 metabolic equivalents), endurance time during constant work rate cycle ergometry to symptom limitation at 75% of the maximum work rate and exertional dyspnea at isotime across the study (aclidinium treatment period only; ITT population).

		**Observations[a]**	**Correlation**	**P-value**
Change from baseline in daily step count[b]	Change from baseline in endurance time (s)	85	0.109	0.319
	Change from baseline in duration of at least moderate activity (min)[b]	85	0.694	<0.0001
Change from baseline in duration of at least moderate activity (min/day)[b]	Change from baseline in endurance time (s)	85	0.022	0.844
Change from baseline in endurance time (s)	Change from baseline in exertional dyspnea at isotime	109	-0.489	<0.0001

[a]Number of observations used to calculate the correlation.

[b]Patients with ≥5 days with data.

ITT, intent-to-treat.
